# Gene specific overwriting of epigenetic signatures to modulate the expression of selected tumor-promoting genes in cancer

**DOI:** 10.1186/1756-8935-6-S1-P15

**Published:** 2013-03-18

**Authors:** Fahimeh Falahi, Christian Huisman, Elisa Garcia Diaz, Hinke G Kazemier, Geke AP Hospers, Marianne G Rots

**Affiliations:** 1 Epigenetic Editing, Dept. Medical Biology and Pathology, 9713 GZ Groningen, The Netherlands; 2 Dept. Medical Oncology, 9713 GZ Groningen, The Netherlands

## Background

Epidermal Growth Factor Receptor-2 (HER-2) and Estrogen receptor-alpha (ER-α) have been found to be dysregulated in several types of cancer. Their dysregulation is associated with aberrant epigenetic modifications. Additionally, expression of ER-α and HER-2 is inversely correlated; their crosstalk has been shown to be involved in endocrine therapy resistance mechanisms [1]. Therapies targeting either receptor result in cell proliferation inhibition for some cancer types; the anti-cancer effect might be further optimized to be more efficient and applicable for more cancer types by co-targeting the genes at the DNA level. As epigenetic modifications provide a way to modify expression of genes in a sustained manner we aim to downregulate HER-2 and ER-α by inducing epigenetic silencing marks specifically onto their promoters (Epigenetic Editing [2]). We also aim to discover the possible crosstalk of ER-a with HER-2 and other important genes in cancer.

## Methods and materials

Towards downregulation of HER-2 and ER-α, expression and epigenetic modification status of promoter of these genes were assessed in a panel of cancer cell lines (bisulfite sequencing and ChIP). Designed zinc finger proteins (ZFPs) targeting genes promoters were fused to a transcription repressor domain [SKD, histone methyltransferases (G9a, SUV39H1), or a DNA methyltransferase domain (C141S [3])] and expressed in cancer cells through viral transduction.

## Results

HER2-ZFP fused to G9a or SUV39H1 induced the intended silencing histone methylation marks (H3K9me2, H3K9me3) on the HER-2 promoter. Up to 4-fold induced H3K9me2 mark was associated with up to 54 ± 2.9% downregulation of HER-2 expression (P<0.0001). Of note, downregulation of HER-2 by induced H3K9 methylation mark was associated with inhibition of cell proliferation and clonogenicity. Additionally, up to 50% downregulation of ER-α was obtained by a ZFP specif c to ER-α fused to SKD, G9a, and C141S.

## Conclusions

These results show that targeted induction of epigenetic marks is instructive in downregulation of HER-2 and ER-α expression. We conclude that Epigenetic Editing might provide a novel and synergistic approach to modulate (onco)genes. In addition, investigation of ER-α crosstalk with HER-2 and other genes might be promising to better interfere with pathways involved in cancer.
